# An alternative method for quantifying coronary artery calcification: the multi-ethnic study of atherosclerosis (MESA)

**DOI:** 10.1186/1471-2342-12-14

**Published:** 2012-07-02

**Authors:** C Jason Liang, Matthew J Budoff, Joel D Kaufman, Richard A Kronmal, Elizabeth R Brown

**Affiliations:** 1 Department of Biostatistics, University of Washington, Seattle, WA, USA; 2 Division of Cardiology, Los Angeles Biomedical Research Institute at Harbor-UCLA Medical Center, Torrance, CA, USA; 3 Environmental & Occupational Health Sciences, Medicine, and Epidemiology, University of Washington, Seattle, WA, USA; 4 Vaccine and Infectious Disease and Public Health Sciences Divisions, Fred Hutchinson Cancer Research Center, Seattle, WA, USA

## Abstract

**Background:**

Extent of atherosclerosis measured by amount of coronary artery calcium (CAC) in computed tomography (CT) has been traditionally assessed using thresholded scoring methods, such as the Agatston score (AS). These thresholded scores have value in clinical prediction, but important information might exist below the threshold, which would have important advantages for understanding genetic, environmental, and other risk factors in atherosclerosis. We developed a semi-automated threshold-free scoring method, the spatially weighted calcium score (SWCS) for CAC in the Multi-Ethnic Study of Atherosclerosis (MESA).

**Methods:**

Chest CT scans were obtained from 6814 participants in the Multi-Ethnic Study of Atherosclerosis (MESA). The SWCS and the AS were calculated for each of the scans. Cox proportional hazards models and linear regression models were used to evaluate the associations of the scores with CHD events and CHD risk factors. CHD risk factors were summarized using a linear predictor.

**Results:**

Among all participants and participants with AS > 0, the SWCS and AS both showed similar strongly significant associations with CHD events (hazard ratios, 1.23 and 1.19 per doubling of SWCS and AS; 95% CI, 1.16 to 1.30 and 1.14 to 1.26) and CHD risk factors (slopes, 0.178 and 0.164; 95% CI, 0.162 to 0.195 and 0.149 to 0.179). Even among participants with AS = 0, an increase in the SWCS was still significantly associated with established CHD risk factors (slope, 0.181; 95% CI, 0.138 to 0.224). The SWCS appeared to be predictive of CHD events even in participants with AS = 0, though those events were rare as expected.

**Conclusions:**

The SWCS provides a valid, continuous measure of CAC suitable for quantifying the extent of atherosclerosis without a threshold, which will be useful for examining novel genetic and environmental risk factors for atherosclerosis.

## Background

Coronary artery calcium (CAC) as detected by computed tomography (CT) is a known marker of subclinical atherosclerosis [1,2] and is a powerful predictor of the risk of coronary events [3-16]. Most commonly, the Agatston, mass, and volume scores have been used to quantify the amount of CAC [17-20].

The Multi-Ethnic Study of Atherosclerosis (MESA), a prospective study with 6814 participants free of cardiovascular disease (CVD) at baseline, was designed to include CT scanning at 4 time points (with two scans per individual at each time point). The CVD exclusion was for those with known clinical disease, and based on self-reported information. The CT reading protocol used a conservative algorithm for lesion detection with high specificity for detection of lesions at the sacrifice of sensitivity. Any voxels not meeting the lesion definition in the algorithm were not used in calculating CAC scores. As such, participants early in the disease progression stages with small calcifications that showed up on the CT scan as less than four contiguous voxels or 130 Hounsfield units (HU) may have been classified as having an undetectable level of CAC. Roughly 50% of the participants in the MESA cohort received a zero CAC score while almost certainly many of these have quantifiable, though less substantial, calcified atherosclerotic lesions. This classification as “zero” has a small impact on our ability to identify individuals at high risk of a coronary event since the high threshold is still adequately sensitive for risk prediction; however, having excess zeros due to misclassification adversely impacts our ability to use CAC measures for modeling and understanding subclinical disease extent and progression.

An ancillary study in MESA focused on ambient air pollution exposures in cohort members is studying the effect of pollutant concentrations on progression of atherosclerosis over 10 years of follow-up [21]. While the relationship between air pollution exposure and CAC is a wholly different question not pursued in this study, it was a motivating factor in developing a valid scoring method to quantify CAC extent throughout the range of detected calcium. More specifically, there are multiple reasons to explore new approaches to quantifying CAC in the MESA. First, current scoring techniques ignore much of the information available in the CT scan that may prove useful in identifying calcified lesions. Second, many of the participants with an Agatston Score (AS) of zero may have had CAC present that did not meet the definition for CAC on the image. Therefore, current CAC measures can fall short as measures of subclinical disease burden while very successfully serving as surrogates for risk of clinical events. Third, the high proportion of zero scores complicates analysis of CAC data. We thus propose an alternative score, the spatially weighted calcium score (SWCS), that uses spatial information in the image combined with voxel-specific weights derived from the phantom, thereby making use of more of the information in the CT scans and also providing a continuous measure of CAC.

## Methods

### Data collection

The MESA was established to study the prevalence, progression, and risks of subclinical cardiovascular disease. Details of the study design have been previously published [22]. A cohort of 6814 men and women, aged 45–84, without known clinical cardiovascular disease was recruited between July 2000 and September 2002 from six urban communities (Baltimore City and Baltimore County, Maryland; Chicago, Illinois; Forsyth County, North Carolina; Los Angeles County, California; New York, New York; and St. Paul, Minnesota). Each of the six centers recruited a population-based sample while oversampling blacks, Chinese, and Hispanics based on self-reported ethnicity at time of enrollment. Institutional review boards at each site approved the study, and all participants gave written informed consent. Further documentation regarding data collection and protocols can be found at http://www.mesa-nhlbi.org[23].

### Risk factors

Information about risk factors and demographics was obtained at enrollment and during the baseline examination. Participants were given questionnaires to obtain information about tobacco usage, passive smoke exposure, alcohol use, medical conditions, medical care access, family history of CVD, reproductive history, and medication use and history. Physical activity was measured using a questionnaire from the Cross-Cultural Activity Participation Study; diet was measured using a modified version of the Insulin Resistance Atheroslecrosis study instrument; anger and anxiety were measured using questionnaires administered on the Spielberger trait anger and anxiety scales; depression was measured on the Center for Epidemiologic Studies Depression scale. Height, weight, and waist and hip circumferences were measured. Blood pressure was measured three times using an automated oscillometric sphygmomanometer (Dinamap Pro 1000; Critikon, Tampa, Florida). Blood samples were taken after a 12-hour fast, and were analyzed by a central laboratory at the University of Vermont for many different measurements including total cholesterol, lipids and lipoproteins (including HDL and LDL), insulin resistance, plasma glucose, triglycerides, high-sensitivity C-reactive protein, and creatinine measurements. Diabetes was defined as use of insulin or oral hypoglycemic agents, or fasting glucose of 126 mg/dL or greater. Body-mass index was defined as weight in kilograms divided by the square of height in meters. Documentation further detailing the questionnaire and measurement protocols can be found at http://www.mesa-nhlbi.org[23] and Bild et al. [22].

### Cardiovascular events (follow-up)

We followed all participants for cardiovascular events for an average of 6.0 years. Interviewers called each participant at intervals of 9–12 months to obtain information about new CVD conditions, CVD interventions, hospitalizations, treatments, changes in life habits, and death. Detailed descriptions of MESA events definitions and follow-up procedure have been previously published [11,15]. For our analyses, we separately considered any CHD (definite or probable myocardial infarction, definite CHD death, or definite or probable angina) and hard CHD (definite or probable myocardial infarction or definite CHD death) events. We note that probable angina cases were only classified as a CHD event if accompanied with a revascularization procedure.

### CT scanning

A detailed report of the CT scanning and reading protocol has been previously reported [24]. At the baseline examination, each participant was given two consecutive unenhanced chest CT scans. The Chicago, Los Angeles, and New York sites used electron-beam CT systems (Imatron C150; GE Medical Systems, Milwaukee, Wisconsin), which scan at a slice thickness of 3.0 mm. The Baltimore, Forsyth County, and St. Paul sites used multi-detector CT systems (Lightspeed QXi, Lightspeed Plus; GE Medical Systems, Milwaukee, Wisconsin; Volume Zoom; Siemens, Erlangen, Germany), which scan at a slice thickness of 2.5 mm. To allow for calibration to compensate for inter-scan variability, radiographic phantoms with known densities of calcium hydroxyapatite (0, 50, 100, and 200 mg/mL) were placed beneath the thorax of each participant for each scan. Participants over 100 kg and at sites using multi-detector CT systems were scanned using slightly different settings.

### CT scan reading

Each scan was read by one of two physician readers at the CT reading center (Los Angeles Biomedical Research Institute at Harbor-UCLA, Torrance, California). As each participant received two consecutive scans, the scans were randomized to lessen the possibility of analysts consecutively reading scans from the same participant. Reading was done using an interactive system to identify the phantoms, determine the artery trajectories and classify candidate calcified plaques. For all techniques presented in this paper, the search for calcified lesions was restricted to be within an 8 mm radius of the trajectories defined by the readers. Further details can be found in previously published reports [24].

### Calculation of the Agatston score

After arterial trajectories were determined and a phantom-based adjustment was applied, candidate calcified plaques were identified by the software with the criteria that each plaque be composed of at least 4 contiguous voxels with an attenuation level of 130HU or greater. The analysts then reviewed each candidate plaque and accepted or rejected its classification as calcified plaque.

To calculate the AS, each accepted lesion was assigned a score by multiplying the lesion volume by a coefficient based on its maximum HU (coefficient of 1 if maximum = 130–199, 2 if 200–299, 3 if 300–399, 4 if > = 400). The AS is the sum of the scores across all accepted lesions. As each participant received two scans, for purposes of this study the average of the two Agatston scores are used for each participant. Use of the average of two scans is intended to reduce noise and to be consistent with methods from other MESA papers using the AS [11,15]. Further details on the Agatston score can be found in previously published reports [17,24]. Participants were notified of their scores on the scale of no coronary calcification, less than average, average, and greater than average.

### Calculation of the spatially weighted calcium score

In developing the SWCS, an important property was for the SWCS to quantify CAC in a manner comparable to the AS for those with AS > 0, while providing meaningful non-zero scores for those with AS = 0. Calculation of the SWCS started with the set of voxels identified by the reader as representing the coronary arteries. First, we assigned a weight to each voxel using a weighting function with parameters derived from the scan’s phantom. The goal of the first step was to calibrate and weight each voxel according to the phantom so that scores across the images were comparable. Second, each voxel was then assigned a score depending on the weight assigned to it and its neighbors. The goal of the second step was to use the surrounding information of each voxel to obtain a more accurate value, and to reduce the impact of noise by upweighting those voxels with neighboring voxels that had high attenuation levels and downweighting those whose neighbors had low attenuation levels. The detailed algorithm appears in the Additional file 1: Appendix with illustrations of the weighting scheme.

### Statistical analysis

Distributions of participant characteristics were described using means and standard deviations. Comparisons of these characteristics across groups were made using the *t*-test. Distributions of the CAC scores were described graphically using kernel density estimators and scatterplots with loess smoothers.

We used Cox proportional hazards regression models similar to those used by Detrano et al. [11] to estimate hazard ratios for hard CHD and any CHD events as they relate to the SWCS and AS in all participants and the subset with AS > 0. The models were all adjusted for race, age, sex, smoking, diabetes, cholesterol, blood pressure, lipid-lowering medication use, and antihypertensive medication use. The AS and SWCS were transformed by taking the base-2 logarithm after adding 1 to the score (log_2_[score + 1]).

Kaplan-Meier survival curves for all CHD events were estimated and plotted for both the SWCS and the AS. The AS K-M curves were stratified into the following percentiles: 0-50%, 50–62.5%, 62.5-75%, 75–87.5%, and 87.5-100%. The SWCS K-M curves were stratified similarly, but with additional 0-25% and 25-50% stratifications to further evaluate any additional ordering the SWCS may provide for participants with levels of CAC that are undetectable by the AS.

To summarize the association between the CAC scores and traditionally recognized CHD risk factors, we first summarized the risk factors in a linear predictor (LP) based on MESA data. The LP for each participant was the sum of their covariate values multiplied by the fitted coefficients from a Cox proportional hazards model. The Cox proportional hazards model was fitted using all CHD events as the outcome and baseline age, systolic blood pressure, diastolic blood pressure, total cholesterol, HDL, diabetes, smoking, and sex as covariates. The covariates were chosen based on the Framingham risk variables [25,26]. We used linear regression models to estimate the relationship of the LP with the SWCS and AS in all participants and the subset with AS > 0. The models were all adjusted for weight, height, and site to accommodate different scanners across sites and different settings used for participants over 100 kg scanned using multi-detector CT scanners. The SWCS and AS were transformed by taking the natural logarithm after adding 1 to the score (log[score + 1]).

We used methods similar to those used by Callister et al. [19] to assess the reproducibility of the scores. We calculated the absolute difference between the scores of each scan relative to their mean: $\left|x_{1}-x_{2}\right|/\left[\left(x_{1}+x_{2}\right)/2\right]\times 100$ where *x*_1_ is the score from the first scan and *x*_2_ is the score from the second scan. This was used as the primary measure of reproducibility and referred to as the percent difference for both the SWCS and AS. We also used the intraclass correlation coefficients to assess the reproducibility of the SWCS and the AS. Analyses were all done in R version 2.11.1 [27].

## Results

### Demographics

**Figure 1:**
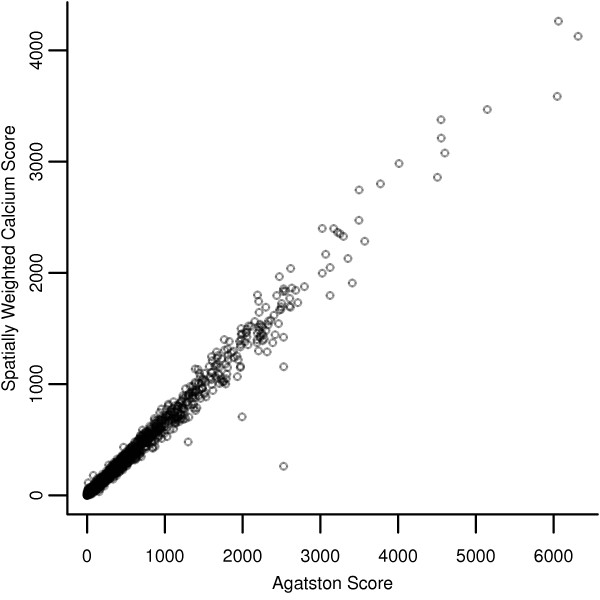
Plot of SWCS against AS for participants with AS > 0 (n = 3269).

We compared demographics and risk factors between those with AS = 0 and AS > 0 (Table 1). The two groups were significantly different with respect to age, sex, hypertension, and treated diabetes, but not for status as a current smoker. We also bifurcated those with AS = 0 into those above and below the median based on their SWCS, comparing demographics and risk factors between these two subgroups. The two subgroups were significantly different with respect to sex, hypertension, treated diabetes, and Framingham 10-year risk, but not for age and current smoking status. As expected, the SWCS of participants with AS = 0 tend to be lower than those with AS > 0, with medians of 0.78 (range, 0.00-284.48) versus 58.99 (range, 0.22-4262.70), respectively. We note that while some participants had very small SWCS, all participants had positive SWCS. A visual representation of the relationship of the AS and SWCS among participants with AS > 0 is shown in the Figure1. As suggested by the scatterplot, there is a high correlation of 0.99 and the linear relationship can be summarized by the equation y=0.52+0.67x.

**Table 1:** Demographics and risk factors

Characteristic	**AS = 0 (n = 3299)**				**AS > 0 (n = 3269)**
	SWCS 0–0.78 <50% tile (n = 1649)	SWCS 0.78-284.48 >50%tile (n = 1650)	P*	All	SWCS 0.22-4262.70
Age (year)	57.88	58.06	0.56	57.97	66.40
Male Sex (%)	30.38% (501)	42.73% (705)	<0.001	36.56% (1206)	57.63% (1884)
Hypertension (%)	30.38% (501)	40.24% (664)	<0.001	35.31% (1165)	54.70% (1788)
Treated Diabetes (%)	4.44% (73)	10.23% (168)	<0.001	7.33% (241)	12.83% (418)
Current Smoker (%)	14.20% (233)	12.78% (210)	0.25	13.49% (443)	12.84% (419)
Framingham 10-yr Risk (Mean [SD])	0.080 (0.062)	0.102 (0.073)	<0.001	0.083 (0.063)	0.137 (0.094)

P-value is for difference between SWCS <50th percentile and SWCS >50th percentile. Calculated using *t*-test for age, and chi-squared tests for other characteristics. Framingham 10-yr Risk calculated using Circulation 1998 method.

**Figure 2:**
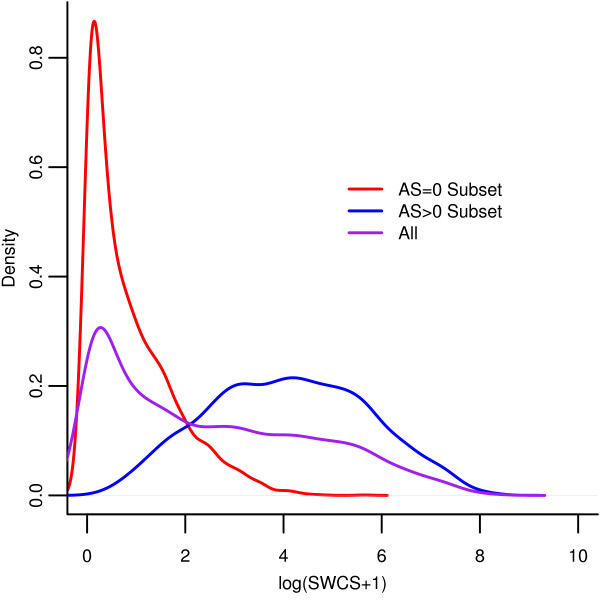
Kernel density estimates of the distribution of the SWCS are shown for AS = 0 (red), AS > 0 (blue) and all (purple).

CT image data was available in 6568 of the 6814 participants. Of these, 6253 (91.8%) had image data available for both CT scans and 315 (4.6%) for one scan. The distribution of the SWCS was quite skewed; thus we present the smoothed density of the SWCS plus a constant of 1, on the natural log scale in Figure2. For the 6568 participants who received at least one scan, we show the smoothed density for all participants, participants with AS > 0, and participants with AS = 0.

### Association of SWCS with CHD events

There were 6508 participants with available measures of all the variables used for the events analysis. The average follow-up time was 6.0 years, during which 291 participants experienced a CHD event of which 171 were hard CHD events. Among those with AS = 0, 34 participants experienced a CHD event, of which 22 were hard CHD events.

An increase in the SWCS was associated with an increase in risk of CHD events (Table 2). A statistically significant relationship was observed when the model was applied to all participants, and when restricted to participants with AS > 0. Furthermore, the relationships were similar to those seen when the SWCS was replaced by the AS in the same models.

**Table 2:** Hazard ratios for hard and all CHD events for twofold increase in AS and SWCSs

		**Hard Coronary Event**		**Any Coronary Event**	
Participants	CAC	No. Events/No. At Risk	Hazard Ratio (95% CI)	No. Events/No. At Risk	Hazard Ratio (95% CI)
All	Log_2_(AS + 1)	171/6508	1.19 (1.14-1.26)	291/6508	1.25 (1.20-1.30)
	Log_2_(SWCS + 1)		1.23 (1.16-1.30)		1.28 (1.22-1.35)
AS > 0	Log_2_(AS + 1)	149/3243	1.16 (1.08-1.25)	257/3243	1.24 (1.17-1.31)
	Log_2_(SWCS + 1)		1.18 (1.09-1.27)		1.25 (1.18-1.33)
AS = 0	Log_2_(SWCS + 1)	22/3265	1.11 (0.79-1.55)	34/3265	1.04 (0.79-1.38)

Each unit increase in Log_2_(Score+1) represents doubling of CAC. All regressions adjusted for race, age, sex, smoking, diabetes, cholesterol, blood pressure, lipid-lowering medication use, and antihypertensive medication use.

A twofold increase in the SWCS or the AS was associated with a 23% (95% CI, 16 to 30) or 19% (95% CI, 14 to 26) increase in risk of hard CHD events, respectively. Furthermore, among participants with AS > 0, a twofold increase in the SWCS or AS was associated with an 18% (95% CI, 9 to 27) or 16% (95% CI, 8 to 25) increase in risk of hard CHD events, respectively.

When we restricted the model to only participants with AS = 0, a twofold increase in the SWCS was associated with a hazard ratio of 1.11 (95% CI, 0.79 to 1.51) for hard CHD events.

**Figure 3:**
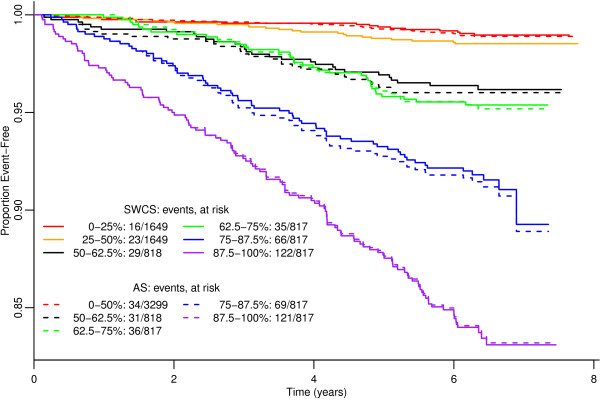
Kaplan-Meier curves stratified by quantiles of the SWCS and the AS, using all CHD events.

The cumulative event rate and number of all CHD events among participants grouped by different percentile stratifications of the scores is shown in Figure3. An ideal risk score would have Kaplan-Meier curves that are well separated and ordered across the quantile-based strata. For the 75–87.5 and 87.5-100 percentile stratifications, the SWCS and AS appear to do equally well at differentiating the quantiles in terms of event risk. The 50–62.5 and 62.5-75 percentile stratifications of the SWCS separate appropriately over time, while those of the AS appear in the incorrect order throughout most of follow-up. That is, that the higher quantile group has lower risk. Finally, the 0–25 and 25–50 percentile stratifications of the SWCS appear to demonstrate some separation, suggesting the SWCS provides a degree of meaningful ordering of CAC in terms of risk in the lower quantiles as well.

### Association of SWCS with CHD risk factors

Among the 6510 participants with complete data for the risk factors, an increase in the SWCS was associated with an increase in the LP (Table 3). For all participants, on the log(CAC + 1) scale, each unit increase in the SWCS and AS was associated with an increase in the mean of the LP of 0.23 (95% CI, 0.22 to 0.24) and 0.18 (95% CI, 0.17 to 0.19), respectively. For participants with AS > 0, on the log(CAC + 1) scale, each unit increase in the SWCS and AS was associated with an increase in the mean of the LP of 0.18 (95% CI, 0.16 to 0.20) and 0.16 (95% CI, 0.15 to 0.18), respectively. For participants with AS = 0, each unit increase in the log of the SWCS plus one was associated with an increase in the mean of the LP of 0.18 (95% CI, 0.14 to 0.22).

**Table 3:** Results of linear regression of linear predictor on SWCS and AS

**Participants**	**CAC**	**Slope**	**95% CI**	**P**	**R**^**2**^
All	Log(AS + 1)	0.182	(0.174-0.190)	<0.001	0.32
	Log(SWCS + 1)	0.226	(0.216-0.235)	<0.001	0.32
AS > 0	Log(AS + 1)	0.164	(0.149-0.179)	<0.001	0.19
	Log(SWCS + 1)	0.178	(0.162-0.195)	<0.001	0.19
AS = 0	Log(SWCS + 1)	0.181	(0.138-0.224)	<0.001	0.12

All regressions adjusted for site, weight, and height.

**Figure 4:**
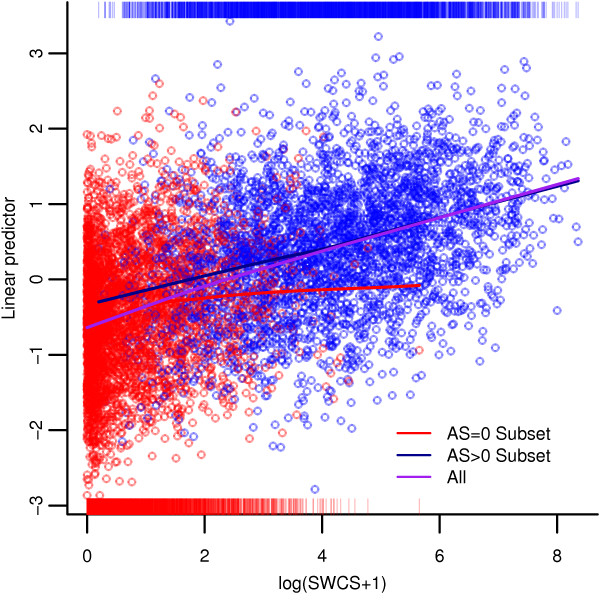
**Scatterplot of linear predictor against SWCS.** This figure plots the linear predictor against the log(SWCS +1). Red circles indicate data points for those participants whose AS = 0, and blue indicates those whose AS > 0. Smoothed curves are also presented to further illustrate the association between these two measures for AS = 0 (red), AS > 0 (blue) and all (purple). The red and blue vertical lines at the bottom and top of the plot indicate the distributions of the log(SWCS +1) for participants with AS = 0 and AS > 0, respectively.

For all participants, and participants with AS > 0, the R^2^ values for the SWCS and AS are comparable. For participants with AS = 0, the R^2^ value is 0.12. A scatterplot of the LP against the log(SWCS + 1) for all participants is shown in Figure4. The dot colors represent participants with AS = 0 or AS > 0. Smoothed curves through the points for all participants, and the subsets of participants with AS = 0 and participants with AS > 0 are also shown in different colors. The vertical lines at the bottom and top of the plot are visual aids indicating the distributions of log(SWCS + 1) for participants with AS = 0 or AS > 0.

### Reproducibility

After excluding scans judged by the scan readers to have unacceptable levels of noise, there were 3102 participants who had at least one scan with AS > 0. The median percent difference across participants was 16.87% and 18.29% for the SWCS and AS, respectively (Table 4). The median of the percent difference in AS minus the percent difference in SWCS was 2.02% (95% CI, 1.66 to 2.40; P < 0.001).

**Table 4:** Reproducibility of SWCS and AS

	**Participants with AS > 0 for at least one scan (n = 3102)**		**Participants with AS > 0 for both scans (n = 2849)**	
Variable	Median	Mean	Median	Mean
% Change in SWCS	16.87	31.23	15.30	24.71
% Change in AS	18.29	41.52	16.26	27.38
% Change in AS minus	2.02 (1.66-2.40)*	10.31 (9.05, 11.56)*	1.05 (0.74-1.51)*	2.68 (1.99-3.36)*
% Change in SWCS				

*P<0.001. P-values based on paired *t*-test for mean and Wilcoxon test for median.

When we performed the same analysis on the subset of 2849 participants with AS > 0 for both scans, the SWCS still showed better reproducibility compared to the AS. We also performed the same analysis further restricted to participants with AS < 50 (n = 892) for both scans and AS < 100 (n = 1307) for both scans. Among these subsets of participants with low but non-zero AS, SWCS still showed better reproducibility.

For the participants for whom we had CT image data for both scans (n = 6253), the intraclass correlation coefficients for the SWCS and AS were 0.988 and 0.989, respectively. For those with AS > 0 for both scans (n = 2849), the intraclass correlation coefficients for the SWCS and AS were both 0.986. For those with AS = 0 for both scans (n = 3151), the intraclass correlation coefficient for the SWCS was 0.406.

## Discussion

In this paper, we present a method for quantifying coronary artery calcification as measured by CT that is independent of a threshold and instead calibrated to the phantom where the attenuation levels represent known densities. We adjust for noise in an automated way using spatial information in the image. The SWCS is shown to be highly related to both risk of CHD events and traditional CHD risk factors. In fact, it retains its strong relationship to traditional risk factors even when the AS equals zero.

Historically, interest in CAC has largely focused on its ability as a predictor of clinical CHD events, and the AS is an effective method of quantifying CAC for these purposes. However, our approach is motivated by studies where the primary interest in CAC is its ability to measure atherosclerotic burden across all levels of disease. In these cases, the AS is less suitable. Specifically, a continuous score for CAC would be more tractable in situations where the extent of atherosclerosis itself is of primary interest.

The primary goal of our analysis was to validate the effectiveness of the SWCS at quantifying CAC. Unfortunately, the “true” burden of CAC is unknown in the MESA patients. Instead, we examined the association of the SWCS with CHD events and known risk factors (via a linear predictor).

In validating the new score, we had three objectives: 1. Ensure that the new score does not lose any information about subclinical disease and risk of CHD events that the AS provides. 2. Determine that a positive score given to participants with AS = 0 is not merely noise, but rather measures lower levels of true subclinical disease. 3. Determine that the new score is at least as reproducible as the AS. We next address how we assessed these three points.

For Point 1, we compared the relationship of the AS and events to that of the SWCS and events. We observed statistically significant and strong relationships between the SWCS and CHD events and risk factors. For the subset of participants with AS > 0, the relationships between the LP and risk of events and CAC scores was similar for both CAC scores, suggesting that the SWCS is replicating the information in the AS in those participants with AS > 0. Furthermore, a simple visual examination of the plot (Figure1) of each non-zero AS against its corresponding SWCS suggests very strong correlation between the two scores. For all participants, and participants with AS > 0, the R^2^ values for the SWCS and AS are comparable. We emphasize that since the goal of our analysis is validation of the SWCS, the focus should be on R^2^ as a descriptive statistic comparing the SWCS and AS rather than as a measure of predictive accuracy. Nonetheless, the relatively low R^2^ values make sense, as the linear regression model only includes CAC, site, weight, and height. In particular, when we consider that CAC has been demonstrated to provide additional predictive information in addition to the traditional cardiovascular risk factors [8,10,15,16,28,29] (which were used to construct the linear predictor), it is unsurprising that the R^2^ values are not high.

Examining these relationships in participants with AS = 0 addresses Point 2. For all CHD events, a doubling of SWCS was associated with a hazard ratio of 1.11 (95% CI, 0.79 to 1.55). For CHD risk, we observed a significant association between the SWCS and the LP; each unit increase in the log(SWCS + 1) was associated with an increase in the mean LP of 0.18 (95% CI, 0.14 to 0.22). The Kaplan-Meier curves for the first two quartiles of the SWCS show a distinct separation. Furthermore, the 50–62.5 and 62.5-75.0 percentile curves for the SWCS appear to be better separated than those of the AS. This suggests that the SWCS algorithm was in fact detecting additional useful information, and that the continuous scale represents a meaningful ordering of CAC suitable for measuring atherosclerotic burden and the attendant risk of CHD events.

To address Point 3, we assessed the reproducibility of the SWCS in comparison with the AS. Using the percent difference as a measure of reproducibility, we found that the median percent difference was 16.87% and 18.29% for the SWCS and AS, respectively. The intraclass correlations were 0.988 and 0.989 for the SWCS and AS, respectively. Our results suggest that the SWCS is at least as reproducible as the Agatston score.

There are two main limitations for the SWCS. One limitation was the lack of a true gold standard for validating the SWCS. The MESA study is composed of a large population of asymptomatic individuals and thus cardiac catheterization (with either angiography or intravascular ultrasound) was not a practical validation method, as it is restricted to studies of symptomatic populations or in treatment trials [18,30-32]. Of course, histologic validation [1,2] was not an option either. We instead used prediction of events and risk of events as a surrogate for a true gold standard, which in itself was limited by the small number of events in those participants with AS = 0. Furthermore, the emergence of models combining CAC measures with traditional risk summaries such as the Framingham risk [28,29] suggests potential for using alternative, potentially richer models for validation. Regardless of the validation used, it would be useful to perform further validation using an independent but comparable dataset. In particular, separate in vitro experimental studies to validate SWCS would be highly desirable. Another limitation involves the applicability of the results outside of the MESA. The SWCS was designed to be a cost-effective approach to rescoring the MESA CT images in a less conservative manner than the original approach to reduce the number of false negatives (when the CAC score is zero but subclinical disease is in fact present). To use the approach presented here in other large studies where comparable calibration phantoms were not scanned or tracing the coronary arteries was not done is not possible.

## Conclusions

In conclusion, we have developed a new continuous method of quantifying coronary artery calcium that may provide a continuous and improved measure of atherosclerosis compared to existing coronary artery calcium scoring methods. This new method is anticipated to be advantageous in research applications, especially in the evaluation of genetic and environmental risk factors.

## Competing interests

The authors declare that they have no competing interests.

## Authors’ contributions

CJL performed the statistical analysis and drafted the manuscript. MJB provided clinical expertise and contributed to the design of the reproducibility analysis. JDK provided epidemiological expertise and contributed to the overall design and coordination of the study. RAK provided input on the technical development of the score and helped with the design of the validation analysis. ERB conceived the study, led its overall design and coordination, and helped to draft the manuscript. All authors read and approved the final manuscript.

## Pre-publication history

The pre-publication history for this paper can be accessed here:

http://www.biomedcentral.com/1471-2342/12/14/prepub

## Supplementary Material

Additional file 1 **Appendix.** For a description of the spatially weighted calcium score in further detail. Click here for file
