# Supplementary material for: An alternative method for quantifying coronary artery calcification: the multi-ethnic study of atherosclerosis (MESA)
Source: BMC Med Imaging. 2012 Jul 2;12:14. doi: 10.1186/1471-2342-12-14 (PMC3443418; doi:10.1186/1471-2342-12-14)
Supplement: Additional file 1 — Appendix. For a description of the spatially weighted calcium score in further detail. [file 1471-2342-12-14-S1.pdf]

Appendix

Step 1 Suppose there are m voxels and a_i represents the attenuation level of the i th voxel. Each voxel is assigned a weight, $w(a_i)$, using a continuous weighting function (Fig A1). The weighting function is based on the mean and standard deviation of the phantom HUs for each scan:

$$w(a_i) = \Phi(a_i; \mu_{100} + 2\sigma_{100}, \sigma_{100}^2)$$

where $\Phi(x; \mu, \sigma^2)$ is the cumulative normal distribution with mean μ and variance σ^2 evaluated at x, and μ_{100} and σ_{100}^2 are the sample mean and variance, respectively, of the HU of the voxels representing the phantom cylinder with 100 mg/mL calcium hydroxyapatite density. The individual weighting functions from a sample of 300 scans are shown in Figure A2. As seen in the plot, the voxels across all scans are placed on the same scale from 0 to 1 using a smooth function. The varying scales and locations of each scan's function reflect the varying phantom HU measurements across all scans. The choice of weighting function is based on a recommendation by McCollough et al (1), who suggested that using scan-specific thresholds based on measurements of the 100mg/mL phantom instead of a uniform 130 HU threshold results in more accurate scores. We do not use a threshold in our algorithm, but instead derive the scan-specific parameters of each weighting function based on the measurements of the 100mg/mL phantom.

During the reading of the CT images, readers marked out sets of voxels that met the thresholding criteria to be potential lesions but were clearly not calcified plaques. We assigned weights of zero to those sets of voxels.

Step 2 Next, we assign a score to each voxel that rescales its own weight based on the weights of its neighbors. We define a voxel to be a neighbor if it is horizontally or vertically adjacent within a slice. We do not consider potential neighboring voxels in adjacent slices. If there are m voxels across all the CT slices and we let $w(a_i)$ be the weighted HU of the i th voxel, its spatially weighted voxel score s_i will be:

$$s_i = w(a_i) \times \sum_{k=1, k \neq i}^{m} w(a_k)\delta_{ik}$$

where δ_{ik} is 1 if the i th and k th voxel are neighbors and 0 otherwise.

Step 3 Finally, the SWCS for the scan of a particular participant is the sum of the new voxel scores of all the voxels:

$$SWCS = \sum_{j=1}^{m} s_j$$

The resulting SWCS is a continuous value that provides additional ordering to participants who otherwise would have received a zero AS. As each participant received two scans, for purposes of this study the average of the two SWCSs are used for each participant unless otherwise noted. Figure A1 illustrates an instance where the SWCS algorithm uses information that would otherwise be ignored by the MESA implementation of the AS algorithm.

References

1. McCollough CH, Ulzheimer S, Halliburton SS, Shanneik K, White RD, Kalender WA. Coronary Artery Calcium: A Multi-institutional, Multimanufacturer International Standard for Quantification at Cardiac CT. Radiology 2007;243:527-538.

A

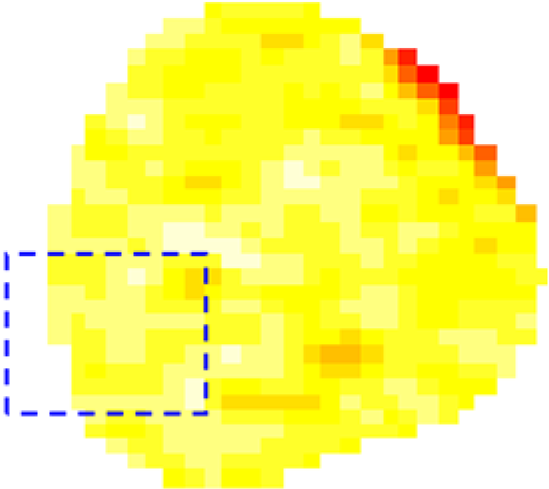

B	1	2	3	4	5	6	7	8	9	10
1			0	46	58	123	133	104	65	7
2			19	25	20	125	163	83	-22	-134
3			127	99	65	104	139	57	-51	-102
4			123	88	112	140	104	57	46	34
5			122	31	71	121	93	83	101	72
6			97	24	59	99	68	27	22	52
7				22	29	72	80	54	28	72
8				17	-20	15	42	61	80	83
9				66	14	-3	11	54	110	148
10				121	127	102	103	93	118	167

C	1	2	3	4	5	6	7	8	9	10
1			0	0.02	0.03	0.21	0.24	0.14	0.04	0
2			0	0	0	0.21	0.34	0.08	0	0
3			0.22	0.13	0.04	0.14	0.26	0.03	0	0
4			0.21	0.10	0.17	0.27	0.14	0.03	0.02	0.01
5			0.20	0.01	0.05	0.20	0.11	0.08	0.13	0.06
6			0.12	0	0.03	0.13	0.05	0	0	0.02
7				0	0.01	0.06	0.07	0.02	0.01	0.06
8				0	0	0	0.01	0.03	0.07	0.08
9				0.04	0	0	0	0.02	0.16	0.29
10				0.20	0.22	0.14	0.14	0.11	0.19	0.36

D	1	2	3	4	5	6	7	8	9	10
1			0	0	0.01	0.11	0.18	0.07	0.02	0
2			0	0	0	0.15	0.28	0.04	0	0
3			0.07	0.05	0.02	0.11	0.17	0.01	0	0
4			0.11	0.05	0.08	0.18	0.10	0.01	0	0
5			0.07	0	0.02	0.11	0.05	0.02	0.02	0.01
6			0.03	0	0.01	0.04	0.02	0	0	0
7				0	0	0.01	0.01	0	0	0.02
8				0	0	0	0	0	0.02	0.05
9				0.01	0	0	0	0.01	0.10	0.18
10				0.05	0.12	0.05	0.04	0.05	0.17	0.27

Figure A1. Step-by-step visualization of SWCS algorithm. A, sample image of an artery from one slice of a scan; B, original HU values of voxels in dotted box with values >130 shaded; C, recalibrated values using phantom data (results from Step 1); D, new smoothed s_i values (results from Step 2). Note that the AS would effectively assign zeroes to these voxels.

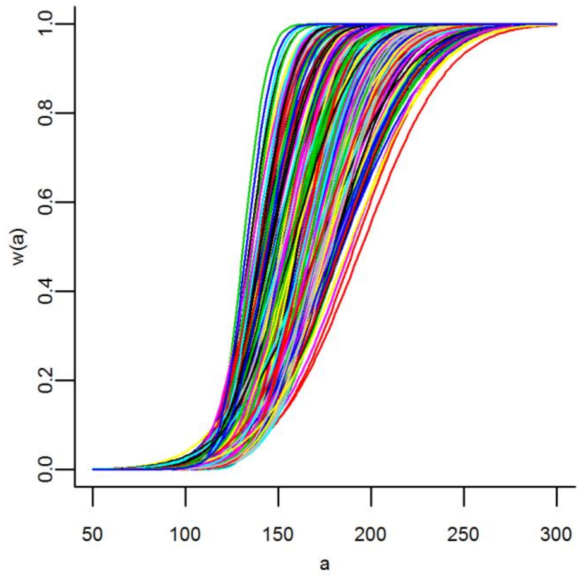

Figure A2. Plot of the individual weighting functions for a sample of 300 scans, where *a* represents the original HU value and *w(a)* represents the corresponding weight.
